# Actigraphic Wake after Sleep Onset and Symptom Severity Correspond with Rumination in Trauma-Exposed Individuals

**DOI:** 10.3390/brainsci13010139

**Published:** 2023-01-13

**Authors:** Fini Chang, Erin C. Berenz, Olusola Ajilore, Scott A. Langenecker, Helen J. Burgess, K. Luan Phan, Heide Klumpp

**Affiliations:** 1 Department of Psychiatry, University of Illinois at Chicago, Chicago, IL 60612, USA; 2 Department of Psychology, University of Illinois at Chicago, Chicago, IL 60607, USA; 3 Department of Psychiatry and Behavioral Health, The Ohio State University, Columbus, OH 43210, USA; 4 Department of Psychiatry, University of Michigan, Ann Arbor, MI 48109, USA

**Keywords:** rumination, worry, trauma, actigraphy, sleep, perseverative cognitions

## Abstract

Rumination and worry are forms of repetitive negative thinking (RNT) commonly associated with internalizing psychopathologies, although less is known about RNT in trauma-exposed individuals with internalizing psychopathologies. Separate lines of research show RNT also plays a role in problematic sleep, which is frequently experienced after trauma exposure. To address gaps in the literature, the current study examines the impact of sleep and symptoms on RNT in trauma-exposed participants. A transdiagnostic sample of 46 unmedicated treatment-seeking trauma-exposed participants completed standard measures of rumination and worry, as well as clinical measures that assessed posttraumatic stress, depression, and anxiety severity. Actigraphic sleep variables were sleep duration, wake after sleep onset (WASO), and sleep efficiency. Sleep and clinical measures were submitted to multiple regression analyses with rumination and worry as dependent variables. The regression results showed that rumination was significantly explained by WASO and posttraumatic stress symptom (PTSS) severity, and the omnibus test was significant. Depression, anxiety, and other estimates of sleep were not significant. No significant results emerged for worry. Preliminary findings suggest that PTSS and WASO, an index of fragmented sleep, may contribute to rumination, but not worry, in trauma-exposed individuals. Longitudinal studies are needed to determine potential causal relationships.

## 1. Introduction

Many in the United States have experienced a traumatic event (i.e., 89.7%) [1], and those who engage in rumination (e.g., dwelling on negative mood or events) [2] and/or worry (e.g., dwelling on potential threat) [3] are at risk of negative outcomes. Negative outcomes include problems with recovery from trauma (e.g., elevated posttraumatic stress symptoms; PTSS) and comorbid anxiety and depression [4,5]. These forms of repetitive negative thinking (RNT) are an attempt to regulate emotions [6]; however, positive associations between RNT and PTSS, anxiety, and depression highlight the transdiagnostic and maladaptive nature of rumination and worry [7,8,9,10]. While the mechanisms of RNT remain to be established, accrued data indicate that deficiencies in control processes (e.g., inhibition) underlie RNT [11,12,13,14], which has implications for the role of sleep in RNT.

Insufficient sleep negatively impacts control processes, and sleep loss (e.g., insomnia) is frequently observed in trauma-exposed individuals [15,16,17,18,19,20,21]; thus, problematic sleep is expected to contribute to RNT. However, relatively little is known about the intersection of sleep, rumination, and worry in trauma-exposed individuals, although accrued findings point to sleep–RNT associations. For example, in adults, greater childhood maltreatment was shown to correspond with worse self-reported sleep quality, and rumination mediated the relationship [22]. It is not clear if objective sleep assessed with wrist actigraphy correlated with rumination or if worry impacted the childhood trauma–sleep quality association as these relationships were not examined [22]. In combat veterans, worse self-perceived sleep positively correlated with PTSS and worry, albeit as a means of distraction, namely dwelling on other worries when experiencing unpleasant or unwanted thoughts. In this case, rumination was not examined [23]. Outside of studies involving trauma exposure, there is evidence of links between RNT and sleep. For example, college students with elevated levels of rumination reported shorter sleep duration and delayed sleep [24]. Additionally, in a prospective study, poor sleep quality in students was predicted by rumination, and this relationship was amplified in students with elevated worry [25]. In a wrist actigraphy study that examined sleep in the context of a psychosocial stressor, rumination in students predicted longer actigraphy-derived sleep onset latency, and latency was longer in students who endorsed both greater rumination and stressor-specific rumination [26]. Worry was not the focus of the study; therefore, its association with actigraphy-based sleep measures is unknown. Even so, findings indicate that actigraphy is sensitive to individual differences in RNT.

Collectively, findings largely based on rumination- or worry-specific studies in disorder-specific cohorts or convenience samples indicate that trauma exposure and problematic sleep play a role in RNT. To address important gaps in the literature, the objective of the current study was to take a transdiagnostic approach and evaluate the extent to which objective estimates of sleep assessed with wrist actigraphy and trauma exposure relate to rumination and worry in participants with internalizing psychopathologies. We hypothesized rumination and worry would be explained by worse sleep. We also expected positive associations between RNT and symptom severity (e.g., PTSS, depression, anxiety severity), but we did not have specific hypotheses regarding symptom dimensions given our transdiagnostic approach and the insufficient literature on the topic.

## 2. Materials and Methods

### 2.1. Participants

This is a secondary data analysis drawn from a clinical trial designed in accordance with the NIMH Research Domain Criteria (RDoC) initiative (ClinicalTrials.gov Identifier: NCT01903447). Participants were not pre-selected for trauma exposure or sleep difficulties. The study protocol and consent were approved by the local Institutional Review Board, and consent was obtained from all participants. A master’s-level or PhD/MD clinician performed psychiatric interviews [27] and interview-based clinical measures. Participants were eligible if they were 18 to 65 years old, had at least one internalizing condition, and reported a total score of ≥23 on the self-report Depression, Anxiety, and Stress Scale (DASS-21) [28], which is based on a dimensional framework of psychopathology. A cut-off point of ≥23 is indicative of a level of symptom severity that warrants treatment [28].

Exclusion criteria included current treatment (e.g., medication or psychotherapy), lifetime history of psychosis (e.g., bipolar disorder, schizophrenia), active suicidal ideation, cognitive dysfunction (e.g., traumatic brain injury, pervasive developmental disorder), and current substance dependence (within the past 6 months). All participants were free of major medical and neurological illnesses as confirmed by a board-certified physician.

Participants were monetarily compensated for their time and all procedures complied with the Helsinki Declaration.

Data were limited to pre-treatment measures in participants who elected to wear an actigraph device and experienced at least one traumatic event based on the Trauma History Screen (THS) [29]. The THS is a self-report measure that evaluates exposure to significant stressors and persisting posttraumatic distress, and it has adequate psychometric properties, including strong convergent validity with posttraumatic stress disorder (PTSD) symptoms [29].

Hypotheses were tested in 46 out of 49 participants; one participant was excluded from analysis due to an actigraph technical or human error, and two participants did not complete RNT measures. With regard to diagnoses, even though the study was designed to be transdiagnostic, information as to whether participants met the diagnostic criteria for a principal diagnosis was obtained. Principal diagnoses included generalized anxiety disorder (*n* = 20), major depressive disorder (*n* = 14), social anxiety disorder (*n* = 6), PTSD (*n* = 3), panic disorder (*n* = 2), and persistent depressive disorder (*n* = 1). Comorbidity was permitted; see Table 1. The majority of participants were female (*n* = 33; 71.7%), the overall age was *M* = 30.02, *SD* = 9.71 years, and education level in years was *M* = 16.80, *SD* = 3.26. As for race and ethnicity, 78.3% (*n* = 36) were Caucasian, 13.0% (*n* = 6) were African American, 2.2% (*n* = 1) were Asian, 2.2% (*n* = 1) reported more than one race, 4.3% (*n* = 2) endorsed “other or unknown”, and 13.0% (*n* = 6) were Hispanic or Latino.

### 2.2. Measures

#### 2.2.1. Posttraumatic Stress, Depression, and Anxiety

The PTSD Checklist-Civilian Version (PCL-C) assessed posttraumatic stress symptom (PTSS) severity. The PCL-C is a 17-item self-report measure that evaluates symptoms over the past month regarding an individual’s self-identified “worst” traumatic event [30]. It is valid and reliable for the screening of PTSD in clinical and community settings (Cronbach’s αs 0.85 to 0.94) [31,32]. For depression and anxiety, the interviewer-based Hamilton Depression Rating Scale (HAMD) and Hamilton Anxiety Rating Scale (HAMA) were used, respectively [33,34].

#### 2.2.2. Repetitive Negative Thinking

The ruminative response scale (RRS) was used to assess rumination [35]. It is a 22-item self-report measure that employs a Likert scale to evaluate the tendency to respond to negative mood with a focus on self, symptoms, and potential consequences and causes of negative mood (Cronbach’s αs 0.88 to 0.92) [36]. For worry, the Penn State Worry Questionnaire (PSWQ) was used [37]. It is a 16-item self-report measure that uses a Likert scale to evaluate the tendency, intensity, and uncontrollability of worry (Cronbach’s αs 0.88 to 0.95) [37].

#### 2.2.3. Sleep

Actigraphy involved participants wearing an actigraph device (15 s epochs; Actiwatch Spectrum, Respironics, Bend, OR) on their non-dominant wrist for 7 days/7 nights continuously, pressing the event marker on the device before and after sleep, and completing simultaneous sleep logs to inform actigraphy data processing. Data were analyzed using the Actiware 6.0.9 Respironics program. Default software settings were used (10 immobile or mobile minutes for sleep onset or offset and a wake activity count threshold of 40), combined with a standardized approach to check the setting of nightly rest intervals, which were guided by event markers, sleep logs, light data, and activity levels [38]. The following validated sleep variables were used [38]: total sleep time (TST; number of minutes scored as sleep in each rest interval); wake after sleep onset (WASO; number of minutes of all wake epochs between sleep onset and offset); and sleep efficiency (proportion of time from sleep onset to offset in each rest interval scored as sleep). Sleep onset latency was also collected; however, it was not analyzed due to its reduced reliability [39]. All variables were scored for each 24 h period and a mean was computed.

### 2.3. Clinical and Sleep Characteristics

Table 1 shows trauma type and frequency. Posttraumatic stress symptoms were in the mild to severe range (PCL-C; *M* = 45.41, *SD* = 14.88) [30], as was anxiety (HAMA; *M* = 17.93, *SD* = 7.81) [33]. Depression symptoms were in the mild to moderate range (HAMD; *M* = 12.91, *SD* = 4.37) [34]. In this sample, PCL-C α = 0.91, HAMD α = 0.68, HAMA α = 0.82, RRS α = 0.90, and PSWQ α = 0.64.

For actigraphy, the average number of days/nights that participants wore the device was *M* = 7.02, *SD* = 1.18, and the number of completed sleep logs was 82.6% (*n* = 38). When assessing for normality of distributions, WASO was positively skewed, and efficiency was negatively skewed. Therefore, log transformation was applied to WASO, and for efficiency, reflection and log transformation were performed, followed by backward transformation to aid interpretation. The analyses and results for WASO and efficiency were based on the transformed data.

### 2.4. Statistical Analyses

Multiple regression analysis (simultaneous entry) was performed with bootstrapping (based on 1000 samples) to evaluate the stability of results. In one model, rumination was the dependent variable (DV) and in another, worry was the DV. For both models, independent variables (IVs) comprised actigraphy-derived sleep measures (TST, WASO, efficiency) and clinical measures, including posttraumatic stress (PCL-C), depression (HAMD), and anxiety (HAMA). All IVs were standardized (z-scored) as the units of measurement differed. For collinearity to be acceptable, tolerance was required to be >0.20 [40,41].

All analyses were two-tailed with an alpha level of 0.05, performed in the Statistical Package for the Social Sciences (SPSS; Chicago, IL, USA; Version 27).

## 3. Results

Collinearity was not acceptable when WASO and sleep efficiency were in the same model (i.e., tolerance < 0.20). Pearson’s correlation verified a strong relationship between these variables (*r* = −0.89, *p* < 0.001); therefore, separate analyses were performed for WASO and sleep efficiency.

### 3.1. Rumination

**Figure 1:**
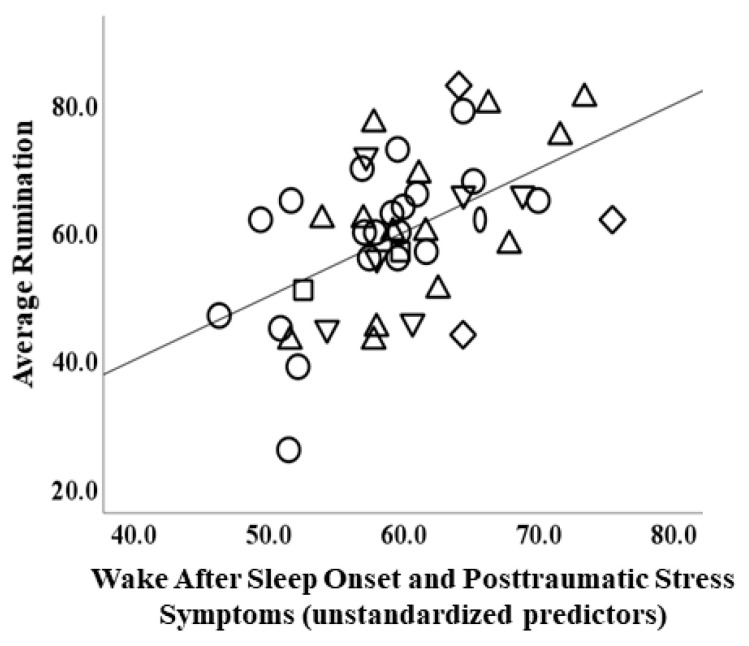
Scatterplot depicting relationship between actigraphic wake after sleep onset and posttraumatic stress symptoms (PTSD Checklist-Civilian Version) (unstandardized predictors) and rumination (ruminative response scale). *Note: circle = principal generalized anxiety disorder; upright triangle = principal major depressive disorder; oval = principal panic disorder; square = principal persistent depressive disorder; diamond = principal posttraumatic stress disorder; downward triangle = principal social anxiety disorder*.

With rumination as the DV and IVs consisting of TST, WASO, and clinical measures (PCL-C, HAMD, HAMA), bootstrapped results showed a greater WASO (B = 3.40, *s.e.* = 1.55, *p* = 0.036) and greater PCL-C (B = 5.24, *s.e.* = 2.08, *p* = 0.018) corresponding with rumination, and the omnibus test was significant [*R*^2^ = 0.28, *F*(5, 40) = 3.16, *p* = 0.017; tolerance > 0.60]. All other IVs were not significant (lowest *p* = 0.49). See Figure 1 for rumination related to WASO and PTSS.

When the analysis was repeated with sleep efficiency and TST in the model, findings were similar for clinical measures, as PCL-C remained the only clinical measure that corresponded with rumination (B = 5.19, *s.e.* = 2.08, *p* = 0.018); all other measures were not significant (lowest *p* = 0.071), although the omnibus test remained significant [*R*^2^ = 0.27, *F*(5, 40) = 2.88, *p* = 0.026; tolerance > 0.60]. 

### 3.2. Worry

With worry as the DV and IVs consisting of TST, WASO, and clinical measures (PCL-C, HAMD, HAMA), bootstrapped results revealed more TST corresponded with worry (B = 2.69, *s.e.* = 1.35, *p* = 0.043). All other IVs were not significant (lowest *p* = 0.74), and the omnibus test was not significant [*R*^2^ = 0.21, *F*(5, 40) = 2.14, *p* = 0.079; tolerance > 0.60].

When the analysis was repeated with sleep efficiency and TST in the model, none of the IVs were significant (lowest *p* = 0.70), and the omnibus test was not significant [*R*^2^ = 0.21, *F*(5, 40) = 2.15, *p* = 0.078; tolerance > 0.60].

Because TST was significant in one model but not the other, post-hoc analysis was performed with TST alone and the bootstrapped result was marginal (B = 2.49, *s.e.* = 1.27, *p* = 0.054). 

## 4. Discussion

The objective of the current study was to determine whether repetitive negative thinking (RNT) was predicted by sleep in trauma-exposed, treatment-seeking individuals, while taking into account symptoms (posttraumatic stress, depression, anxiety). Regression results revealed that more wake after sleep onset (WASO) and greater posttraumatic stress symptom (PTSS) severity each corresponded with rumination. No other significant findings were observed for rumination, and no significant results were detected for worry, although more total sleep time was marginally associated with worry.

The findings partially support the hypotheses. As expected, worse sleep positively corresponded with rumination, although the results were limited to WASO, an index of fragmented sleep, which reduces the efficiency of sleep [42]. Therefore, preliminary findings suggest that disruption in sleep maintenance may contribute to rumination. Because rumination is associated with impairment in control processes (e.g., inhibition, attention, working memory) [11,12,13,14], and insufficient sleep is associated with impairment in control processes [15], it is possible that a more fragmented sleep enhances engagement in rumination by further decreasing control processes. However, as we did not collect measures of control processes, it is important for future studies to test this conjecture.

With regard to clinical characteristics, PTSS significantly explained variance in rumination when taking anxiety, depression, and sleep measures into account. The findings extend that of prior reports which indicate that rumination positively relates to posttraumatic stress symptom severity in trauma-exposed individuals [8,43]. A possible mechanism could involve the stress system; for example, trauma-exposed individuals experience more stress than healthy individuals, which may be due to changes in the stress response system [44]. Therefore, we speculate that the robust relationship between rumination and trauma symptoms may reflect attempts to regulate negative mood magnified by, or related to, stress, although this remains to be tested.

No significant relationships between worry and sleep were observed, although the result for total sleep time was marginal and in an unexpected direction as the relationship was positive. Because sleep quantity does not necessarily represent sleep quality, as evidenced by the low agreement between actigraphy and self-perceived sleep [45,46,47,48,49], the findings may relate to subtle disturbances in sleep that are not sensitive to actigraphy. It will be important to replicate these findings in a larger sample and assess for sleep quality before drawing conclusions. Lastly, we may have been underpowered to detect PTSS effects, as the effect size between PTSS and worry is relatively small (i.e., *r* = 0.29) [8].

### Limitations

In addition to issues concerning power, other limitations include the cross-sectional design of the study and the lack of subjective sleep measures. Additionally, few participants met the full criteria for PTSD; therefore, the results are more consistent with a dimensional, rather than categorical, nosology of PTSD [50]. Still, the findings have clinical implications as individuals with subthreshold PTSD experience distress and impairment [51]. Another limitation is the lack of a comparative healthy control group, problematic sleep group without psychopathology, and trauma-exposed group without psychopathology. The majority of the participants were relatively young, female, and Caucasian, and depression was in the mild–moderate range; thus, the results may not generalize to samples with different demographic or clinical characteristics. We did not screen for sleep disorders (e.g., insomnia, hypersomnia, obstructive sleep apnea) or measure or instruct participants to refrain from substances that may impact sleep (e.g., caffeine, alcohol), which may have introduced confounds. A portion of participants (i.e., 17.4%) did not complete a sleep log; hence, sleep onset/offset could not be confirmed in these participants, and we could not verify that the participants adhered to the instructions on completing sleep logs. Actigraphy involved 15 s epochs, and limited research suggests that this time window is consistent with “gold standard” polysomnography sleep measures [52], but earlier studies have been validated with 30 s epochs [53]. There was no direct manipulation of sleep (i.e., sleep deprivation in a controlled setting); therefore, the results rely on estimates of naturalistic sleep. Additionally, negative thoughts that facilitate pre-sleep arousal (e.g., sleep-related worry), which impedes sleep [54], were not assessed. Lastly, information regarding the participants’ weekday/weekend schedules or chronotype were not obtained; therefore, we cannot rule out the possibility that this may have impacted the findings.

## 5. Conclusions

The preliminary results suggest that more fragmented sleep and greater posttraumatic stress symptom severity contributes to rumination in trauma-exposed individuals with internalizing psychopathologies. The findings may represent a vicious cycle where problematic sleep and trauma-related symptoms increase rumination, which in turn worsens sleep and trauma symptoms, although longitudinal studies are required to test this supposition. No significant sleep or clinical measures corresponded with worry, highlighting differences between rumination and worry. The findings suggest that improving fragmented sleep and trauma-related symptoms may reduce rumination in trauma-exposed individuals, even if they do not meet the criteria for PTSD. Relatedly, it is important to consider the potential impact of sleep and trauma exposure in interventions that target RNT (e.g., emotion regulation therapy) [55].

## Figures and Tables

**Table 1:** Distribution of principal diagnosis and comorbidity; trauma event type based on Trauma History Screen.

Principal Diagnosis	*n*	%
Generalized anxiety disorder	20	43.5
Major depressive disorder	14	30.4
Social anxiety disorder	6	13.0
Posttraumatic stress disorder	3	6.5
Panic disorder	2	4.3
Persistent depressive disorder	1	2.2
**Comorbidity**	***n***	**%**
Social anxiety disorder	18	39.1
Major depressive disorder	14	30.4
Generalized anxiety disorder	12	26.1
Panic disorder	10	21.7
Posttraumatic stress disorder	8	17.4
Specific phobia	7	15.2
Persistent depressive disorder	5	10.9
Obsessive compulsive disorder	2	4.3
Alcohol abuse	1	2.2
Substance abuse	1	2.2
**Trauma Event Type**	***n***	**%**
Sudden death of close family or friend	19	41.3
Suddenly abandoned by spouse, partner, parent or family	13	28.3
Some other sudden event that made you feel very scared, helpless or horrified	10	21.7
Hurricane, flood, earthquake, tornado or fire	10	21.7
Forced or made to have sexual contact (adult)	9	19.6
Hit or kicked hard enough to injure (child)	8	17.4
Sudden move or loss of home and possessions	7	15.2
Seeing someone die suddenly or get badly hurt/killed	6	13.0
Really bad car, boat or airplane accident	6	13.0
Forced or made to have sexual contact (child)	4	8.7
Hit or kicked hard enough to injure (adult)	3	6.5
Really bad accident at work or home	3	6.5
Attacked with gun, knife or weapon	2	4.3
During military service—seeing something horrible or badly scared	2	4.3

## Data Availability

The data and material that support the findings of this study are available from the corresponding author upon reasonable request.
